# Publisher Correction: Single-cell analysis of childhood leukemia reveals a link between developmental states and ribosomal protein expression as a source of intra-individual heterogeneity

**DOI:** 10.1038/s41598-021-85034-7

**Published:** 2021-03-02

**Authors:** Maxime Caron, Pascal St-Onge, Thomas Sontag, Yu Chang Wang, Chantal Richer, Ioannis Ragoussis, Daniel Sinnett, Guillaume Bourque

**Affiliations:** 1 Department of Human Genetics, McGill University, Montréal, Québec Canada; 2 McGill Genome Centre, Montréal, Québec Canada; 3 CHU Sainte-Justine Research Center, Montréal, Québec Canada; 4 Department of Pediatrics, University of Montreal, Montréal, Québec Canada; 5 Canadian Center for Computational Genomics, Montréal, Québec Canada

Correction to: *Scientific Reports* 10.1038/s41598-020-64929-x, published online 15 May 2020

This Article contains errors, where incorrect versions of Table 1 and Table 2 have been typeset due to a technical error. The correct Tables 1 and 2 appear below.

**Table 1:** A correct version of the original Table 1.

Sample_name	Tissue	Class	Diagnosis	Subtype	Percentage_blasts	dna_index	Age_at_diagnosis_months	Gender	Karyotype
ETV6-RUNX1_1	bmmc	Pediatric	B-ALL	ETV6-RUNX1	95	1	145	Male	49,XY,t(6;12)(?q15;?q13),+10,+16,+21 or der(21)t(12;21)(p13;q22)[15]/50,idem,+22[3]/46,XY[2]
ETV6-RUNX1_2	bmmc	Pediatric	B-ALL	ETV6-RUNX1	93	1	47	Male	46,XY,del(11)(q14.1)[2]/46,idem,der(5)dup(5)(p12p15.33)del(5)(q14.3q35.3),del(12)(p11.23)[2]/46,XY[11]
ETV6-RUNX1_3	bmmc	Pediatric	B-ALL	ETV6-RUNX1	75	1	27	Male	47,XY,t(12;21)(p13.2;q22),+der(21)t(12;21)(p13.2;q22)[6]/46,XY[37]
ETV6-RUNX1_4	bmmc	Pediatric	B-ALL	ETV6-RUNX1	89	1	56	Male	45,XY,-9,der(12)t(9;12)(q21.11;p11.22),der(18)t(17;18)(q21.31;q21.2)[8]/46,XY[12]
HHD_1	bmmc	Pediatric	B-ALL	HHD	79	1.15	67	Male	50~54,XY,+X,+Y,+4,+8,+14,+17,+18,+21[8]/46,XY[3]
HHD_2	bmmc	Pediatric	B-ALL	HHD	84	1.13	60	Male	53,XY,+X,+4,+6,+14,+18,+21,+21[19]/46,XY[1]
PRE-T_1	bmmc	Pediatric	T-ALL	pre-T	86	1	44	Male	46,XY[20]
PRE-T_2	bmmc	Pediatric	T-ALL	pre-T	54	1	135	Male	46,XY,del(6)(q13q22)[10]/46,XY[11]
PBMMC_1	bmmc	Pediatric	Healthy control	Control	NA	NA	23	Male	NA
PBMMC_2	bmmc	Pediatric	Healthy control	Control	NA	NA	53	Male	NA
PBMMC_3	bmmc	Pediatric	Healthy control	Control	NA	NA	31	Male	NA

**Table 2:** A correct version of the original Table 2.

Sample	Number of cells	Mean reads per cell	Median genes per cell	Number of reads	Sequencing saturation	Reads mapped confidently to genome	Fraction reads in cells	Total genes detected	Median UMI per cell
ETV6-RUNX1_1	2,776	76,003	1,830	210,987,037	84.0%	92.0%	89.8%	18,397	5,312
ETV6-RUNX1_2	6,274	52,662	1,239	330,404,706	87.9%	90.5%	85.1%	20,005	2,435
ETV6-RUNX1_3	3,862	85,994	1,181	332,110,056	90.3%	85.3%	80.8%	19,909	2,815
ETV6-RUNX1_4	5,069	59,411	1,304	301,155,525	84.3%	83.5%	88.3%	19,414	3,940
HHD_1	3,728	83,388	1,579	310,871,911	78.3%	89.1%	77.1%	19,061	4,712
HHD_2	5,013	64,741	2,059	324,551,184	73.4%	87.1%	93.3%	20,798	5,643
PRE-T_1	2,959	109,972	2,114	325,408,071	80.5%	85.3%	74.9%	19,775	6,309
PRE-T_2	2,748	118,053	1,685	324,412,280	86.1%	86.7%	81.8%	20,568	5,037
PBMMC_1	1,612	303,228	880	488,803,883	NA	NA	78.5%	NA	2,573
PBMMC_2	3,105	101,124	1,091	313,990,607	81.0%	79.2%	79.9%	19,927	6,408
PBMMC_3	2,229	145,472	1,085	324,258,554	91.4%	87.1%	61.6%	18,793	3,008

