# Supplementary material for: Single-cell analysis of childhood leukemia reveals a link between developmental states and ribosomal protein expression as a source of intra-individual heterogeneity
Source: Sci Rep. 2020 May 15;10:8079. doi: 10.1038/s41598-020-64929-x (PMC7228968; doi:10.1038/s41598-020-64929-x)
Supplement: Supplementary file 1 — Supplementary Information. [file 41598_2020_64929_MOESM1_ESM.pdf]

Single-cell analysis of childhood leukemia reveals a link between developmental states and ribosomal protein expression as a source of intra-individual heterogeneity

Maxime Caron[1,3], Pascal St-Onge[3], Thomas Sontag[3], Yu Chang Wang[2], Chantal Richer[3], Ioannis Ragoussis[2], Daniel Sinnett[3,4,*], Guillaume Bourque[1,2,5,*]

[1] Department of Human Genetics, McGill University, Montreal, Quebec, Canada
[2] McGill University and Genome Quebec Innovation Centre, Montreal, Quebec, Canada
[3] CHU Sainte-Justine Research Center, Montreal, Quebec, Canada
[4] Department of Pediatrics, University of Montreal, Montreal, Quebec, Canada
[5] Canadian Center for Computational Genomics, Montreal, Quebec, Canada

[*] Corresponding authors: guil.bourque@mcgill.ca and daniel.sinnett@umontreal.ca

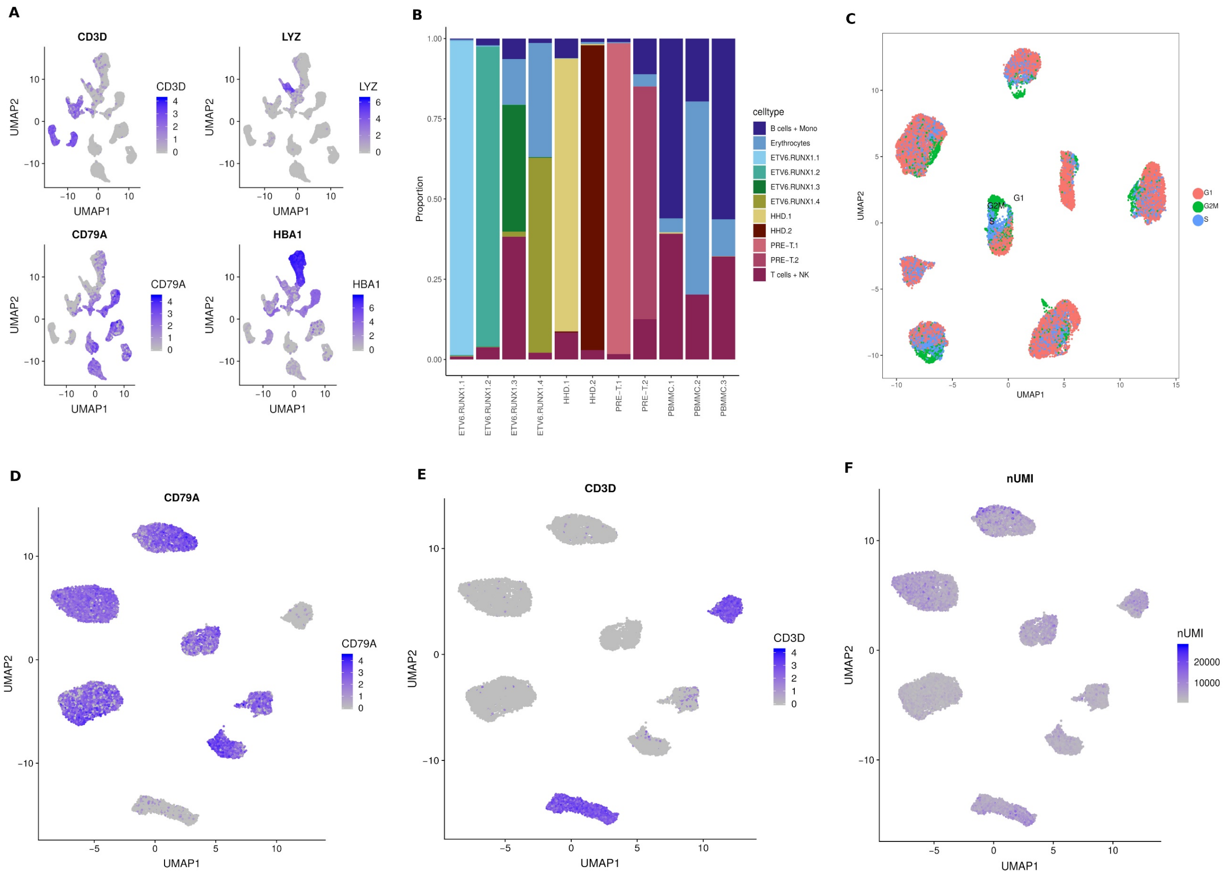

<u>Supplementary Figure 1.</u> Healthy pediatric BMMCs and cALL cells. **A)** Expression of cell type markers in healthy pediatric BMMCs (n=6,836) and cALL (n=32,086). **B)** Proportion of cells from cancer samples clustering with healthy PBMMC cell clusters (T + NK cells, B cells + monocytes, erythrocytes). **C)** Cell cycle phases after regressing out S and G2/M phase scores. **D)** Expression of the *CD79A* B cell marker gene in cancer cells. **E)** Expression of the *CD3D* T cell marker gene in cancer cells. **F)** Number of unique molecular indexes (nUMI) in cancer cells.

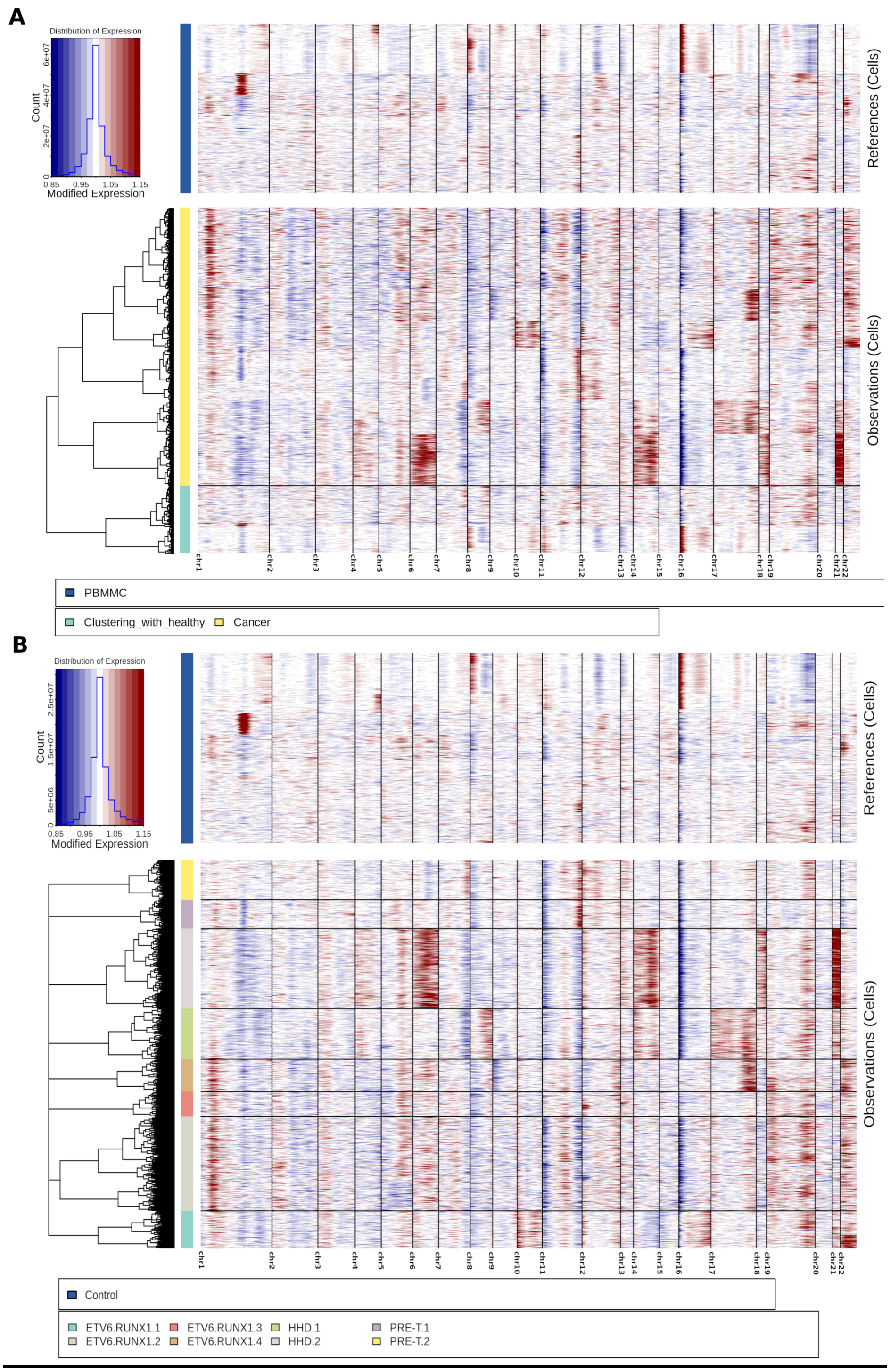

<u>Supplementary Figure 2.</u> Copy number profiles of single cells. A) Copy number profiles of cells from cancer samples clustering with PBMMCs (Clustering_with_healthy) and cells from cancer samples not clustering with PBMMCs (Cancer), using PBMMCs as a baseline reference. **B)** Copy number profiles of cancer cells in **A)** that are in the G1 phase only, using PBMMCs as a control baseline reference.

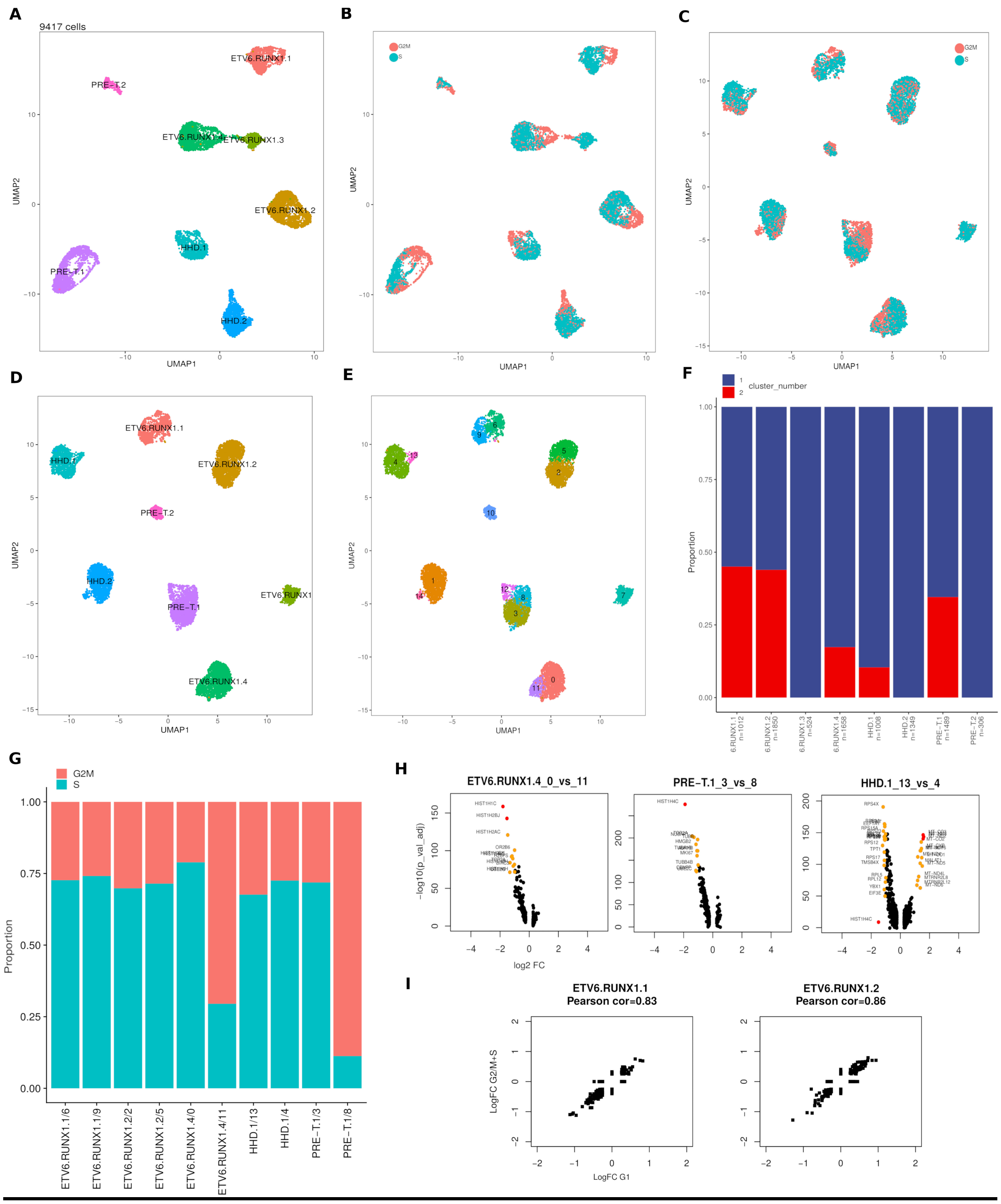

Supplementary Figure 3. Intra-individual cluster identification using cells in S and G2/M cell cycle phases. A) UMAP representation of cells from cancer samples in S and G2/M phases (n=9417). **B)** UMAP representation of cell cycle phases. **C)** UMAP representation of cell cycle phases after regressing out phase scores, which diminishes its effect but does not remove it entirely. **D)** UMAP representation of cells from cancer samples in S and G2/M phases after regressing out phase scores. **E)** Optimal clustering solution identified using the same approach as the one used for cells in G1. **F)** Proportion of cells belonging to each intra- individual cluster after removing clusters having less than 10% of cells per sample. **G)** Proportion of cells in S or G2/M phases per transcriptional cluster, showing unbalanced proportions for some samples. **H)** Differentially expressed genes

between newly identified clusters showing deregulated cell cycle genes (*TOP2A*, *MKI67* and histone (*HIST**)) and mitochondrial genes (*MT-**). **I)** Comparison of log fold changes of differentially expressed genes between clusters identified in both G1 and S+G2/M phases.

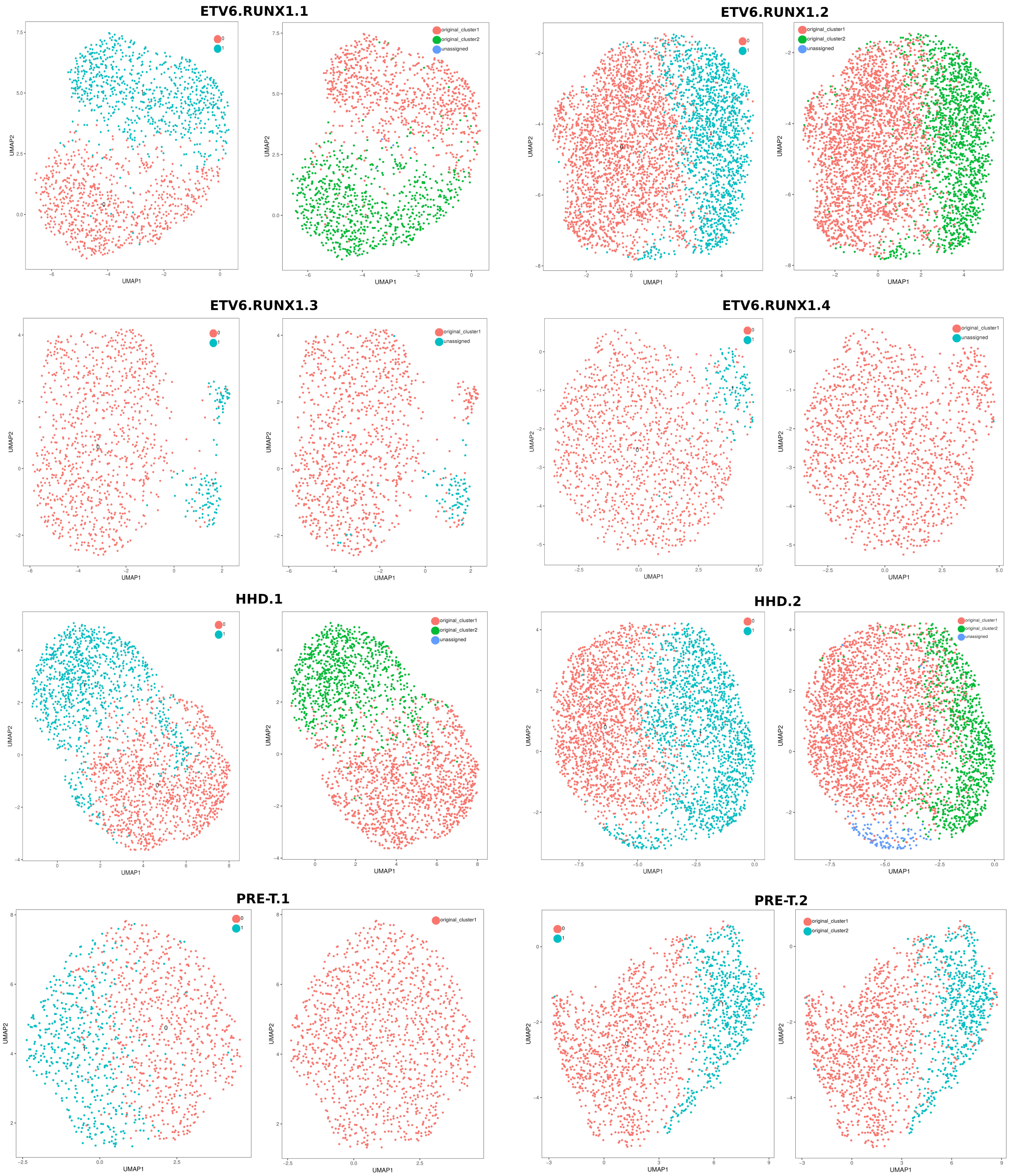

<u>Supplementary Figure 4.</u> Identification of transcriptional clusters in each cancer sample. Intra-individual transcriptional clusters were identified in each individual cancer sample using cells that do not cluster with PBMMCs and that are in G1 phase. The clustering resolution was increased until two clusters were returned. For each sample, cluster labels identified using individual samples (left) are compared to cluster labels obtained using the all samples approach (right), showing good overlap for most samples.

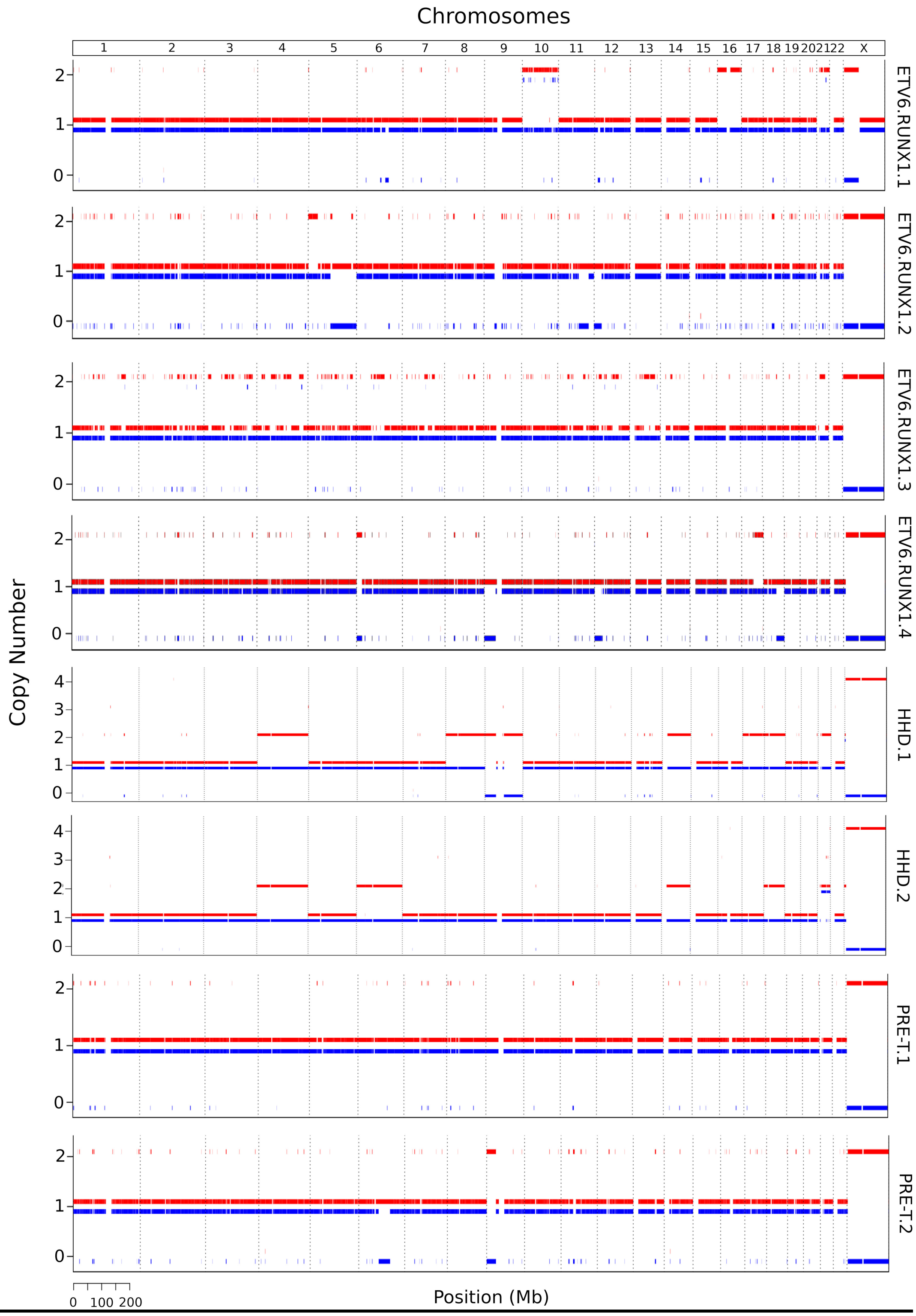

Supplementary Figure 5. Copy number profiles of cALL samples using exome sequencing data.

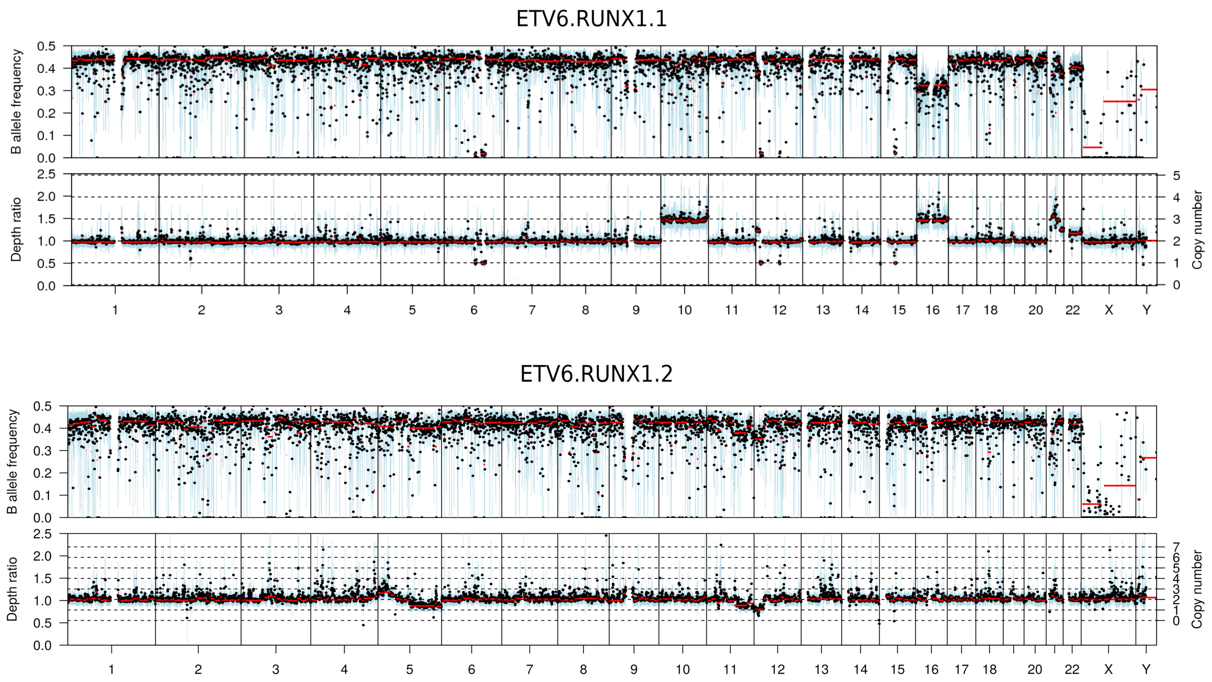

Supplementary Figure 6. B-allele frequency and depth ratio of cALL samples using exome sequencing data.

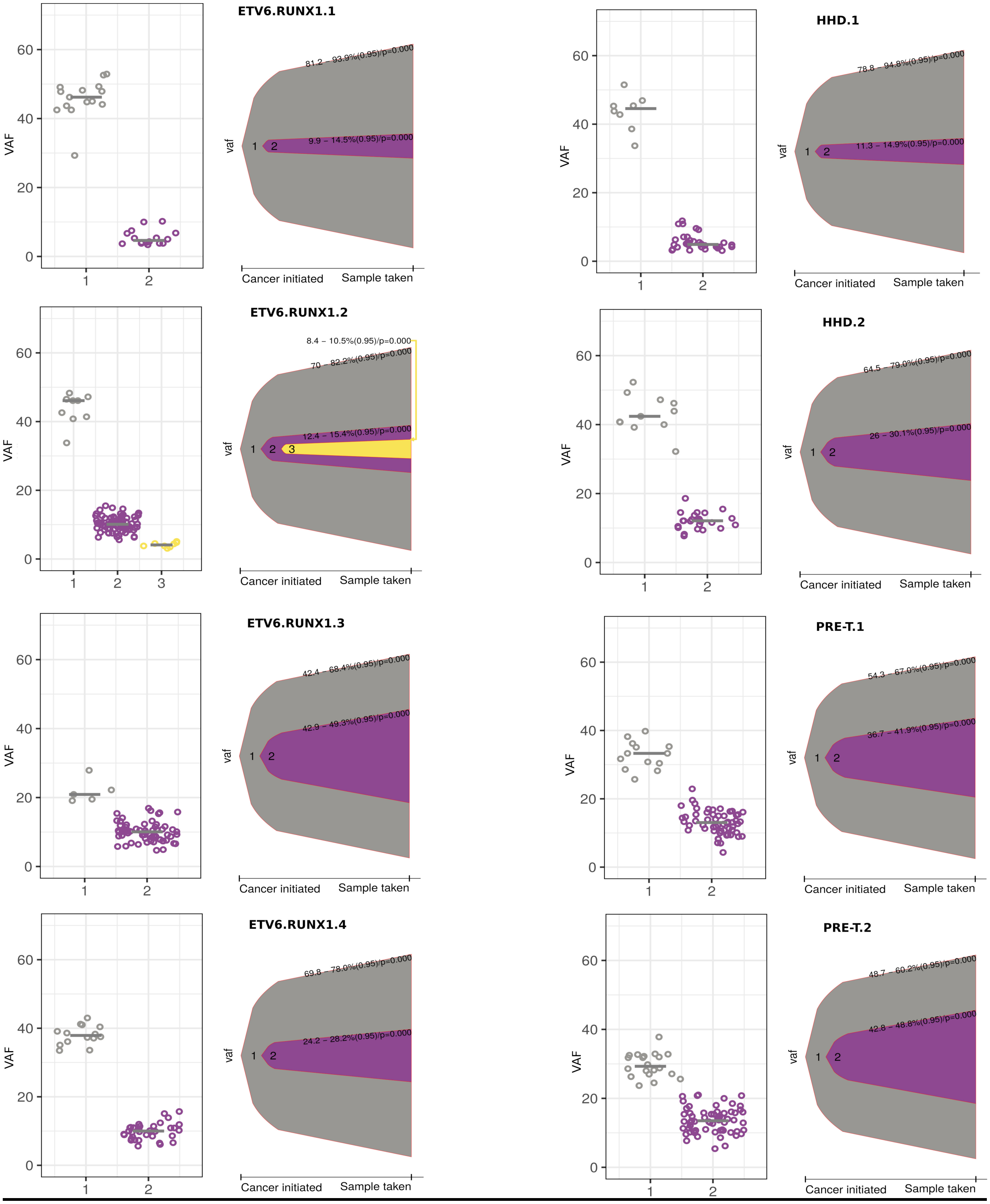

Supplementary Figure 7. Predicted genetic clonal evolution models of cALL.
